# Effects of an interactive web-based support system via mobile phone on preference-based patient participation in patients living with hypertension – a randomized controlled trial in primary care

**DOI:** 10.1080/02813432.2023.2301567

**Published:** 2024-02-07

**Authors:** Hanna Vestala, Marcus Bendtsen, Patrik Midlöv, Karin Kjellgren, Ann Catrine Eldh

**Affiliations:** a Department of Health, Medicine and Caring Sciences, Linköping University, Linkoping, Sweden; b Center for Primary Healthcare Research, Department of Clinical Sciences Malmö, Lund University, Lund, Sweden; c University of Gothenburg Centre for Person-Centered Care, University of Gothenburg, Gothenburg; d Department of Public Health and Caring Sciences, Uppsala University, Sweden

**Keywords:** E-health, hypertension, patient engagement, patient participation, preferences, primary healthcare, self-management

## Abstract

**Objective:**

To estimate the effects of an interactive web-based support system *via* mobile phone on preference-based patient participation in patients with hypertension treated in primary care (compared with standard hypertensive care only).

**Design:**

A parallel group, non-blinded, randomized controlled trial, conducted October 2018–February 2021. Besides standard hypertensive care, the intervention group received eight weeks of support *via* mobile phone to facilitate self-monitoring and self-management, tentatively providing for augmented patient engagement.

**Setting:**

31 primary healthcare centers in Sweden.

**Subjects:**

949 patients treated for hypertension.

**Main outcome measures:**

The effects on preference-based patient participation, that is, the match between a patient’s preferences for and experiences of patient participation in their health and healthcare. This was measured with the 4Ps (Patient Preferences for Patient Participation) tool at baseline, after 8 weeks, and at 12 months. Data were registered electronically and analyzed with multilevel ordinal regression.

**Results:**

At baseline, 43–51% had a complete match between their preferences for and experiences of patient participation. There was an indication of a positive effect by a higher match for ‘managing treatment myself’ at 8-weeks in the intervention group. Such preference-based participation in their health and healthcare was reversed at 12 months, and no further effects of the intervention on preference-based patient participation persisted after 12 months.

**Conclusion:**

The interactive web-based support system *via* mobile phone had a wavering effect on preference-based patient participation. There is a prevailing need to better understand how person-centered patient participation can be facilitated in primary care.

## KEY POINTS

- Although patient participation is essential when having a long-term condition, interventions optimizing individuals’ engagement have not been fully identified.
- About half of the patients with hypertension in this study did not experience participation in the manner and extent they preferred.
- A web-based support system via mobile phone improved some aspects of patient participation in the short- but not long term.
- Strategies to better identify patients’ preferences for patient participation are needed, to evaluate and improve the outcome of care.

## Introduction

As healthcare progresses towards person-centered services, patients are increasingly encouraged to take an active part in their health and healthcare [1,2]. Legislations around the world link patients’ rights to fair conditions for patient participation [3], including the option to engage in one’s health and healthcare in alignment with one’s needs, condition, and preferences. By means of patient participation, a more person-centered and trustworthy healthcare can be accomplished [4].

Despite the established significance of patient participation, conceptual clarity is lacking. The semantical origin of participation is sharing, indicating that patient participation comprises for example communicating knowledge and experiences, learning of, and taking part in plans and goals, and knowing how and when to proceed in self-care [5]. Thus, patient participation exceeds merely consenting to or sharing health decisions and/or adherence to prescribed procedures [1,6]. Rather, patient participation is defined by a reciprocal relationship between the patient and the professional, striving to bridge any breach caused by knowledge and information differences, with opportunities for being engaged and partaking in intellectual and/or physical activities [6,7]. Patient participation thus relies on learning and reciprocity, where patients’ preferences harmonize with their experiences of being engaged, that is, preference-based patient participation [8,9].

When living with a long-term condition, it is important to have opportunities for participation in one’s healthcare [10]: to understand and share one’s experiences, and to engage in self-management and self-care [11]. Hypertension is one such long-term condition, with risks for cardiovascular morbidity and mortality [12]. Approximately 40% of the adult population worldwide suffers from hypertension and to date, most of them do not reach the target blood pressure (BP) (i.e.; <140/90 mmHg measured in a clinical setting or 135/85 mmHg if measured at home) [13]. In Sweden, primary hypertension is normally treated in primary healthcare. The treatment for hypertension often consists of pharmaceuticals, but lifestyle changes have shown significant effects, which calls for a high level of patient participation [14,15]. The treatment is often based on severity of the hypertension, and patients preferences are not always considered [16]. One reason for lack of goal achievement, whether it is reaching the target BP or achieving successful lifestyle modifications, is presumably the limited opportunities for a shared understanding of health needs and everyday commitment to treatment [17,18].

Being involved in self-care when living with hypertension is associated with modifications like reducing salt and alcohol consumption, smoking cessation, increased physical activity, and weight loss. Consequently, coaching and educating patients to normalize BP, as well as promoting life-style modifications are beneficial [13]. This promotes patients to understand how they can affect their BP and take on a more active role in their hypertensive care [18–20]. Furthermore, being able to measure BP at home can increase the patient’s understanding of their hypertension [14,21,22] as can an interactive web-based support system [21–24]. There is limited research with regard to how such interventions affect patients’ participation, particularly preference-based patient participation.

In 2018–2020, the PERHIT (PERson-centeredness in Hypertension management using Information Technology) study was performed across 31 primary healthcare centers (PHCC’s) in Sweden. The PERHIT study investigated whether an additional, interactive, web-based support system *via* mobile phone-intervention (besides regular hypertensive care) reduced BP in patients with hypertension, compared to just regular primary care [25]. Hypothetically, the intervention would empower patients living with hypertension in both understanding and mastering their condition, increasing their potential to participate in health and healthcare issues. In addition, the education, training, and support provided would presumably initiate a more collaborative approach, with the patient’s knowledge and experience recognized by the primary care staff [20].

The objective of this study was to estimate the effects of the interactive web-based support system on preference-based patient participation in patients with hypertension in primary care, in comparison to standard hypertensive care.

## Material and method

A non-blinded randomized controlled trial with parallel groups was conducted, including patients from the 31 PHCC’s taking part in PERHIT [25]. While the primary objective of PERHIT was to study the effects of an interactive web-based support system on BP, which is reported on by Andersson et al. [26], preference-based patient participation represented a secondary outcome. The trial had ethical approval (Dnr. 2017/311 and Dnr. 2019/00036) and was registered with ClinicalTrials.gov: NCT03554382. This manuscript contains relevant items from the CONSORT checklist.

### Settings and participants

Eligible PHCCs in southern Sweden were recruited *via* the unit head for each site. For those complying, a research project team representative held a local information meeting for the nurses and physicians, and training was provided for the web-based interactive system. This included how to introduce the patients in the intervention group to the support system’s content, functions, and usage.

Patients with hypertension, actively treated with at least one anti-hypertensive medicine were approached regarding the PERHIT study by their local nurse or physician during a regular visit. Only patients able to understand spoken and written Swedish and with access to a mobile phone were considered [25]. The information about the trial included contact information for the research project team, with the same information displayed in waiting areas at the PHCCs.

Patients interested in joining the trial signed their informed consent form during the visit or returned this form later. Only patients fulfilling inclusion criteria and providing informed consent were enrolled in the trial. Patients were not financially compensated but the PHCCs were reimbursed for each patient enrolled (due to the extra work for the staff). Following consent, participants were asked to complete a baseline questionnaire in a REDCap® electronic case report form (eCRF) [27], hosted at Clinical Studies Sweden- Forum South, Region Skåne on a computer at the PHCC provided by the PERHIT-trial.

### Randomization and allocation

Randomization was done 1:1 after baseline assessments using block randomization with blocks varying in size between 4 and 6 and stratified for each PHCC. The randomization sequence was created by an independent statistician directly in the eCRF and members of the research team did not have access to the sequence, nor could they manipulate it. A qualified, independent monitor oversaw the data and study process. The eCRF displayed allocation after completion of the baseline questionnaire, making the allocation procedure automatic and not possible to manipulate. Given the intervention, patients knew if they were in the intervention or control group after allocation, as did the staff, that is, the trial was not blinded [26].

### Intervention

The control group received standard hypertensive care while the intervention group, in addition to the same standard hypertensive care, was given access to an interactive support system *via* mobile phone called CQ, (developed by Circadian Questions AB, Sweden) facilitating self-monitoring and self-management and were sent motivational messages [23]. The intervention group patients were instructed by their nurse or physician on how to use the web-based system, as well as how to measure their BP at home. Furthermore, the patients were instructed on how to use the web-based system to report their BP, well-being, symptoms, lifestyle, medication intake, and side effects for eight consecutive weeks. The system also asked questions regarding side effects of the treatment depending on patients’ medication. Patients could choose motivational messages to increase their physical activity, to eat healthily, or reduce stress [20,25,28]. Moreover, the system provided graphs in a secure web portal, based on the patients self-reported information during the 8-weeks of the intervention. This showed how variation in the BP depended on, for example, whether they were physically active or forgot their medicine [25].

All study participants (control and intervention groups) were scheduled for follow up visits at 8-weeks and 12-months post randomization, where follow-up questionnaires were completed on computers provided to the PHCC by the PERHIT trial [20,25,28].

### Outcomes and measures

**Figure 1.**
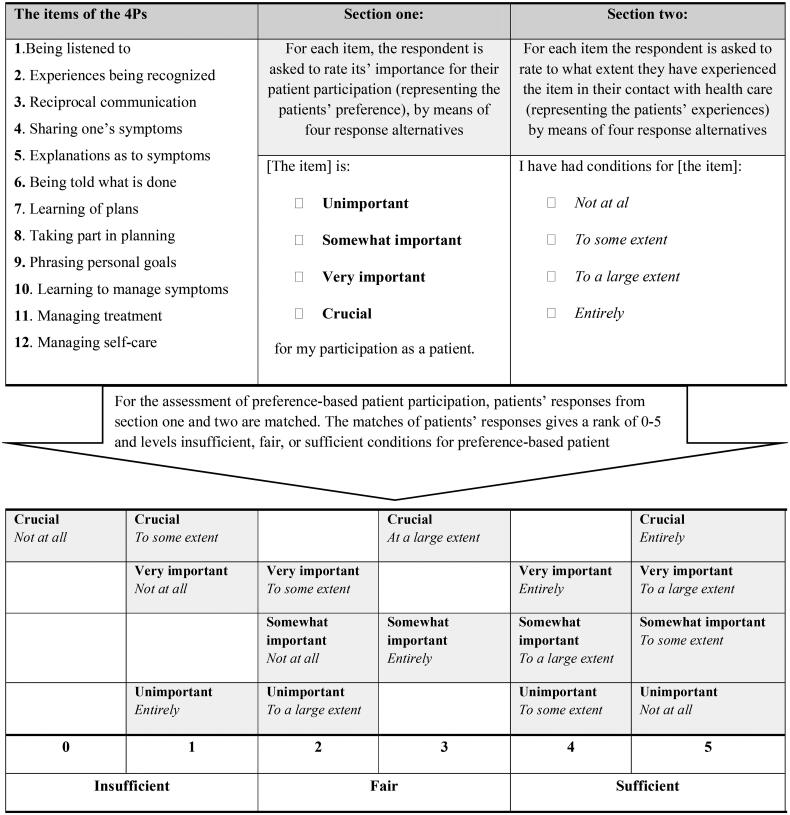
Description of the items and response alternatives in the two sections of the patient preferences for patient participation tool (the 4 Ps) and an overview of the 16 possible combinations between patient preferences for, and experiences of patient participation. Preferences in bold and experiences in italics. Ranks 0–5 and the three levels of preference-based patient participation [29].

The primary outcome of the trial was BP [26], with preference-based patient participation listed as a secondary outcome. The assumption was that the intervention would render the patients enhanced opportunities to engage in their health and healthcare for hypertension [6]. Patients completed the Patient Preferences for Patient Participation tool, the 4Ps three times: at baseline, at the end of the 8-week intervention period, and at the 12-month follow-up. The 4Ps is a recognized and valid instrument, employing 12 items known to conceptualize and exhaust patient participation, as listed in Figure 1 [1,5,29].

The outcome of this paper, the preference-based patient participation, is derived from combining each patient’s preferences and experiences per item. With two sections (preferences and experiences, respectively) and four response alternatives per item, there are 16 possible combinations of match or mismatch, see Figure 1. A complete match between preference and experience is considered the best outcome, and the closer to a complete match between preference and experience, the better. Further, hypothetically, more experience of participation than preferred is better than less than preferred. The degree of match is positioned in six ranks, ranging from a complete mismatch between preferences and experiences (0) to a complete match [5], as illustrated in Figure 1. The ranks are also classified into three levels of conditions for preference-based patient participation: 0-1 = insufficient, 2-3 = fair, 4-5= sufficient [29].

### Data analysis

The participants’ data were analyzed in the groups to which they were randomized (intent-to-treat). An available data approach was taken for missing data, assuming that data was missing at random. The effects of the intervention on each of the 12 items were estimated with multilevel ordinal regression, including a group by time coefficient, and adjusted for baseline values of each respective outcome measure. Adaptive intercepts were included for participant and site, and adaptive effects for site. Models were estimated using Bayesian inference with the median of the marginal posterior distributions representing point estimates of effect. The 2.5% and 97.5% quartiles of the marginals were calculated to represent 95% compatibility intervals (CI).

The odds being modelled were those representing having higher match scores, e.g. the odds of having a score of four or greater versus lower scores. The odds ratios should therefore be interpreted as the relative difference in odds of higher scores between groups, all else being equal. The proportional odds assumption implies that this odds ratio is the same between each pair of outcome groups, and graphical tests indicated that this assumption was reasonable (See Appendix 1, supplementary material). Odds ratios above 1.0 (one) indicate increased odds of scoring higher among intervention group participants and vice versa.

**Table 1.** Baseline characteristics of randomized participants.

	Total population *n* = 949	Intervention group *n* = 482	Control group *n* = 467
Characteristics			
Age at inclusion, mean (SD), *range*, median, years	62.9 (9.9), *25–92*, 64	62.8 (9.8), *25–85*, 64	63 (10), *33–92*, 64
Men, n (%)	542 (57.1)	283 (58.7)	259 (55.5)
Years since hypertension diagnosis, mean (SD), median	10.2 (9.8), 7	9.6 (8.8), 7	10.7 (10.7), 7
Education			
Up to high school, n (%)	217 (22.9)	102 (21.2)	115 (24.6)
High school, n (%)	430 (45.3)	216 (44.8)	214 (45.8)
College/university, n (%)	281 (29.6)	150 (31.1)	131 (28.1)
Risk factors			
BMI at baseline, mean (SD)	28.9 (4.5)	28.8 (4.3)	28.9 (4.7)
Smoker, %	47 (5.0)	23 (4.8)	24 (5.1)

## Results

**Figure 2.**
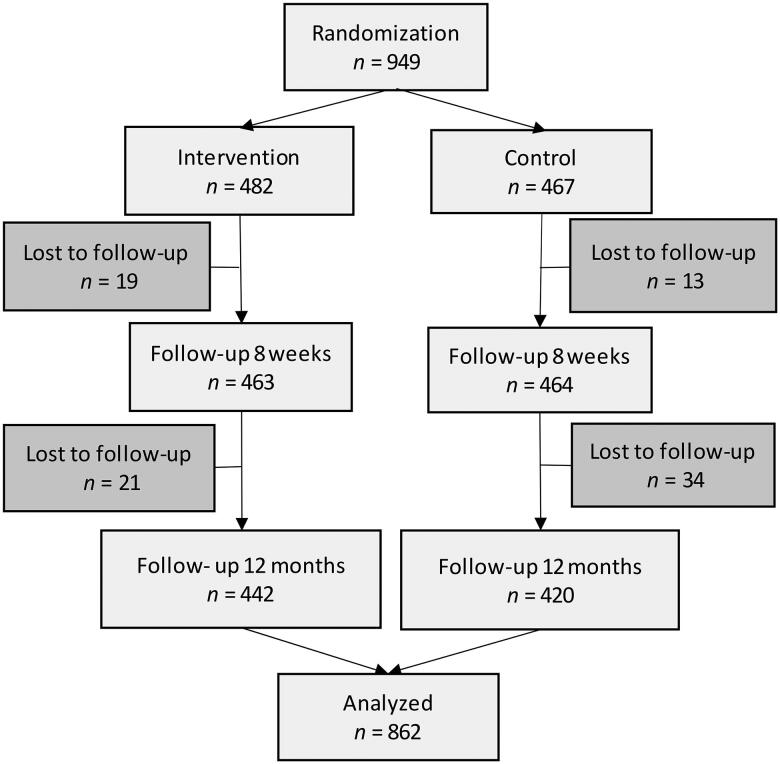
Flow diagram of the PERHIT-trial. Reasons why data were lost to follow-up were patient decision or health care professional decision to not continue or other reasons such as an error in randomization, patient being hospitalized, bad internet connection, mobile phone not working, PHCC not completing the study, not visiting PHCC because of the pandemic, not being able to come to PHCC, were not called, patient deceased, spouse deceased, illness, unknown. PHCC = primary health care center [26].

Patients were randomized into the study starting 2018/10/24, and the last patient completed the 12- month follow-up on 2021/02/02. A CONSORT flow diagram is shown in Figure 2. A total of 949 patients were randomized, 482 to the intervention group and 467 to the control group. For baseline characteristics, please see Table 1. Of the participants in the intervention group, 52.2% were men, versus 47.8% in the control group. The mean age was 63 years (SD 10 years, median 64 years) in both groups. Baseline preference-based patient participation is presented in Table 2. At baseline, about half (44–52%), of the participants had a complete match, rank 5, for preference-based patient participation.

**Table 2.** Baseline data on preference-based patient participation.

Preference-based patient participation for each item of the 4Ps’, at baseline, n (% of total 949 participants)							
	0= insufficient	1= insufficient	2 = fair	3 = fair	4 = sufficient	5 = sufficient	missing
**1**. Being listened to	3 (0.3)	32 (3.4)	71 (7.5)	115 (12.1)	228 (24)	477 (50.3)	23 (2.4)
**2**. Experiences being recognized	2 (0.2)	14 (1.5)	120 (12.6)	77 (8.1)	250 (26.3)	458 (48.3)	28 (3)
**3**.Reciprocal communication	4 (0.4)	43 (4.5)	88 (9.3)	120 (12.6)	233 (24.6)	438 (46.2)	23 (2.4)
**4**. Sharing one’s symptoms	2 (0.2)	46 (4.8)	58 (6.1)	118 (12.4)	214 (22.6)	484 (51)	27 (2.8)
**5**. Explanations as to symptoms	9 (0.9)	73 (7.7)	132 (13.9)	135 (14.2)	141 (14.9)	432 (45.5)	27 (2.8)
**6**. Being told what is done	2 (0.2)	40 (4.2)	89 (9.4)	122(12.9)	206 (21.7)	465 (49)	25 (2.6)
**7**. Learning of plans	8 (0.8)	51 (5.4)	105 (11.1)	130 (13.7)	193 (20.3)	437 (46)	25 (2.6)
**8**. Taking part in planning	6 (0.5)	51 (5.4)	145 (15.3)	101 (10.6)	212 (22.3)	408 (43)	26 (2.7)
**9**. Phrasing personal goals	11 (1.2)	54 (5.7)	141 (14.9)	70 (7.4)	200 (21.1)	433 (45.6)	40 (4.2)
**10**. Learning to manage symptoms	17 (1.8)	63 (6.6)	128 (13.5)	144 (15.2)	129 (13.6)	436 (45.9)	32 (3.4)
**11**. Managing treatment	3 (0.3)	13 (1.4)	39 (4.1)	74 (7.8)	298 (31.4)	487 (51.3)	35 (3.7)
**12**. Managing self-care	12 (1.3)	60 (6.3)	118 (12.4)	69 (7.3)	221 (23.3)	432 (45.5)	37 (3.9)

### Preference-based patient participation

Table 3 displays the estimates of odds ratios of higher rank of preference-based patient participation. There were no marked effects of the intervention on preference-based patient participation as measured with the 4Ps. Relative to one another, the effects on Item 6 (*Being informed of what is being done for me*) stand out, indicating that there was a 93.6% probability that those with the intervention had higher matches than those without at 8-weeks. However, the data suggests that this effect had waned at 12-months. In addition, Item 11 (*Managing treatment*) seems to have benefited those in the intervention group at first, with an 87.7% probability of a positive effect at 8 weeks. However, at the 12-month follow-up, the effect of the intervention on this item had reversed with a 92.6% probability of a negative effect.

**Table 3.** Estimated effect and longevity of the intervention on preference-based patient participation.

	8 weeks post randomization		12 months post randomization	
	Median^a^ (95% CI)	Post. Prob^b^ >/< null	Median^a^ (95% CI)	Post. Prob^b^ >/< null
1. Being listened to	0.94 (0.69; 1.27)	66.1%	0.93 (0.68; 1.28)	67.2%
2. Experiences being recognized	1.04 (0.75; 1.44)	60.2%	1.00 (0.71; 1.40)	50.0%
3. Reciprocal communication	1.10 (0.80; 1.50)	71.6%	0.80 (0.56; 1.12)	90.7%
4. Sharing one’s symptoms	1.02 (0.71; 1.44)	53.2%	1.13 (0.79; 1.64)	74.1%
5. Explanations as to symptoms	1.12 (0.82; 1.51)	75.6%	1.21 (0.88; 1.69)	87.8%
6. Being told what is done	1.27 (0.93; 1.75)	93.6%	0.96 (0.68; 1.35)	58.7%
7. Learning of plans	1.17 (0.86; 1.62)	84.7%	1.17 (0.82; 1.64)	81.4%
8. Taking part in planning	1.07 (0.77; 1.49)	65.3%	1.10 (0.79; 1.57)	72.7%
9. Phrasing personal goals	0.96 (0.70; 1.32)	59.8%	1.06 (0.76; 1.49)	64.6%
10. Learning to manage symptoms	1.19 (0.87; 1.63)	85.8%	1.17 (0.83; 1.63)	81.3%
11. Managing treatment	1.22 (0.87; 1.74)	87.7%	0.75 (0.51; 1.10)	92.6%
12. Managing self-care	1.13 (0.82; 1.59)	77.2%	0.97 (0.68; 1.39)	56.6%

^a^ Median of the marginal posterior distribution of odds ratios given by multilevel ordinal regression adjusted for baseline value with adaptive intercepts for subject and adaptive intercept and effects for site. 95% CI are defined by the 2.5% and 97.5% quartiles.

^b^ The posterior probability that the odds ratio is greater or less than the null (OR = 1) in the direction of the median of the marginal posterior distribution.

## Discussion

We found no evidence of marked effects of the web-based support system on preference-based patient participation in patients living with hypertension. Regarding item 11*(Managing prescribed treatment myself),* the intervention group had a slight advantage after the 8-weeks of the intervention, but at the 12-month follow-up they had a much lower preference-based patient participation compared to the control group. A similar trend was seen for item 12 *(Managing self-care)* as well as item 3 *(Having reciprocal communication).* Better adherence to prescribed treatments often correlates with a reciprocal relationship with healthcare professionals, and how patients experience the care given [30]. Motivational messages and reminders *via* mobile phones have previously been found to double the odds of adherence to treatments [31], although the potential link to patients’ preferences and experiences of participation is unclear.

Fletcher et al. [32] describe how self-monitoring BP helps patients to visualize their BP, set goals, and reach their target BP, which is ultimately achieved by adhering to treatments and a healthy lifestyle. During the 8-weeks of the intervention, the patients in the intervention group received support and encouragement to manage self-care and treatment which may explain the results on item 6 (*Being told what is done).* Further, they were stimulated to actively reflect on the effect their hypertension and daily life has on one another. In a pilot study of PERHIT (with 50 patients), Hallberg et al. [24] found that patients appreciated the feedback given by the web-based system and considered the informative graphs useful when conducting lifestyle changes. When having received the extra support during the intervention, one can expect that the patients sensed a lack thereof after the eight weeks.

The intervention might have had a positive effect on the intervention group in terms of learning symptom management (item 10) and learning of plans (item 7). This would indicate that the intervention held an educational aspect that resonated with the participants, which they could continue to utilize, as suggested by Andersson et al. [20]. Different self-monitoring systems for hypertension are often used in clinical trials or in the development of interventions, but rarely in standard hypertensive practice [32]. Rather, the longevity of the effects of a relatively short of period self-monitoring BP remains to be explored before used on a larger scale.

The 4Ps is suggested to be valid and appropriate for preference-based patient participation [5,29,33] and is increasingly used in Sweden and elsewhere. Compared with previous findings, the patients with hypertension had a similar pattern of preference-based patient participation at baseline as those with a chronic renal disease and dialysis [2], with a relatively low percentage having an optimal match regarding item 8 *(Taking part in planning).* Whether it is agreeable that roughly half of the patients only have opportunities to engage in such a way and to the extent they prefer is debatable. Even when including all patients with sufficient conditions for preference-based patient participation (that is, rank 4 and 5), there is still 20–25% of the patients living with hypertension who had preferences for being engaged that were not matched in primary care at all three time points.

Patient participation has been warranted for decades, although there is a more recent understanding that authentic conditions require a recognition of patients’ preferences, that is preference-based patient participation [6]. Despite the call for more person-centered, integrated care, conditions for preference-based patient participation are not fully achieved. An intervention such as the interactive web-based support system trialed in PERHIT had no substantial effect but still indicates that opportunities for being involved in self-management, learning about one’s symptoms and having regular support are favorable when living with hypertension [20,21,34]. Nevertheless, there is a prevailing need to better understand how better opportunities for person-centered patient participation can be facilitated.

### Limitations

While the large number of participants, representing the conditions for patient participation across 31 primary healthcare centers, is a strength, the outcome measure of patient participation builds on self-reported data. This, in combination with the allocation, induces a risk of bias. For instance, it is possible that patients in the intervention group may have found the support system desirable and therefore altered their responses to both support the system and the research project, and/or thought that the system could be removed if not shown to be working for them. In such a case, this would explain why there was a reversal of effects once the support system was not available (at the 12-month first follow-up). Likewise, the control group patients may have altered their responses to show that they needed further support. It is also possible that the patients’ preferences for participation changed independently of the study they were partaking in; preferences, values, and expectancies are known to represent complex relationships [35].

The self-report nature of the outcome patient participation induces the possibility of assessment reactivity; being presented with attributes to define a common yet broad concept may render new insights. Patients who get such opportunities to reflect on their preferences may alter how they conceptualize and respond to subsequent interactions with healthcare, with a more articulate idea as to how they prefer and expect to be involved in their hypertensive care. Gaining such insights into one’s own preference is hypothesized to be one of the mechanisms by which the support system was intended to work [35]. The assessment with the 4Pss may have activated the same mechanisms in the control group, leading to effect estimates biased towards the null.

## Conclusion

About half the participants reported sufficient preference-based patient participation, that is, a complete match between their preferences and experiences of patient participation. While the interactive web-based self-monitoring system did not conclusively improve preference-based patient participation, especially in the long term, there was evidence that it strengthened patients in learning to manage their symptoms related to hypertension. This indicates a further need for health professionals to recognize patients’ preferences for participation in primary care for and with patients with hypertension.

## Supplementary Material

Supplemental Material

## Practice implications

Healthcare professionals guided by how and to what extent patients’ wish to engage can direct their efforts in accord. For patients with hypertension, this seems an appropriate issue to address, along with managing treatment, symptoms and learning about plans for their treatment.
