# Supplementary material for: Effects of an interactive web-based support system via mobile phone on preference-based patient participation in patients living with hypertension – a randomized controlled trial in primary care
Source: Scand J Prim Health Care. 2024 Feb 7;42(1):225–33. doi: 10.1080/02813432.2023.2301567 (PMC10851821; doi:10.1080/02813432.2023.2301567)
Supplement: Supplemental Material [file IPRI_A_2301567_SM5482.docx]

# **Appendix**

**The multilevel ordinal regression model**

The multilevel ordinal regression model used to analyze 4P match outcomes is presented in Equation 1. The model included adaptive intercepts for subject ($\gamma_{i}$) and site ($\Sigma_{j,1,1}$). In addition, adaptive slopes were used for site ($\Sigma_{j,2,1},\Sigma_{j,1,2},\Sigma_{j,2,2}$). Time by group coefficients were included in the models to estimate effects at the two follow-up intervals. The models also included a covariate for baseline values of each respective outcome measure, treated as a continuous variable.

Covariate coefficients were given Student’s t priors (degrees of freedom = 3, mean = 0, and scale = 2.5). Adaptive intercepts and slopes were given standard normal priors with standard deviations following half-Student’s t priors (degrees of freedom = 3, mean = 0, and scale = 2.5). The correlation matrix ($\Phi$) for site adaptive intercepts and slopes was given Lewandowski, Kurowick, and Joe (LKJ) priors with uniform density over correlation matrices.

**Equation 1: Multilevel ordinal regression model used to analyze 4P match outcomes**

#

$$\begin{matrix} Match\sim& ordered\_logistic\left( \eta,\mathbf{c} \right) \\ \mathbf{c}\sim& StudentT\left( \nu=3,\mu=0,\sigma=2.5 \right) \text{| Cutpoints} \\ \eta= & \gamma_{i}+\beta_{1}\times Group+\beta_{2}\times Time+\beta_{3}\times Group\times Time+\beta_{4}\times Match_{Baseline}+\Sigma_{j,1,1}+\Sigma_{j,2,1}\times Group+\Sigma_{j,1,2}\times Time+\Sigma_{j,2,2}\times Group\times Time \\ \beta_{1:4}\sim& StudentT\left( \nu=3,\mu=0,\sigma=2.5 \right) \\ \gamma_{i}\sim& normal\left( 0,\sigma_{\gamma} \right) \text{| Adaptive intercept for subject} \\ \sigma_{\gamma}\sim& StudentT_{+}\left( \nu=3,\mu=0,\sigma=2.5 \right) \\ \Sigma_{j,1:2,1:2}\sim& normal\left( 0,\sigma_{\Sigma_{1:4}} \right) \text{| Adaptive intercept and slopes for site} \\ \sigma_{\Sigma_{1:4}}\sim& StudentT_{+}\left( \nu=3,\mu=0,\sigma=2.5 \right) \\ \Phi\sim& LKJ\left( \nu=1 \right) \end{matrix}$$

# **Proportional odds assumption**

The ordinal regression models used to estimate effects of the web-based support system on preference-based patient participation require an assumption of proportional odds. It is recommended to test this assumption graphically comparing the odds ratios produced by estimating multiple logistic regression models, one for each value of the ordinal variable. Supplementary Figures 1 to 24 show graphical comparison of odds ratios for each item at each follow-up interval. When the proportional odds assumption holds, the symbols align along the Yes/No lines for group. Overall, it was found that the proportional odds assumption was reasonable for all items.

|   **Figure 1 - Proportional odds for Item 1 at 8-weeks follow-up** |
| --- |
|   **Figure 2 - Proportional odds for Item 1 at 12-month follow-up** |
|   **Figure 3 - Proportional odds for Item 2 at 8-weeks follow-up** |
|   **Figure 4 - Proportional odds for Item 2 at 12-month follow-up** |
|   **Figure 5 - Proportional odds for Item 3 at 8-weeks follow-up** |
|   **Figure 6 - Proportional odds for Item 3 at 12-month follow-up** |
|   **Figure 7 - Proportional odds for Item 4 at 8-weeks follow-up** |
|   **Figure 8 - Proportional odds for Item 4 at 12-month follow-up** |
|   **Figure 9 - Proportional odds for Item 5 at 8-weeks follow-up** |
|   **Figure 10 - Proportional odds for Item 5 at 12-months follow-up** |
|   **Figure 11 - Proportional odds for Item 6 at 8-weeks follow-up** |
|   **Figure 12 - Proportional odds for Item 6 at 12-months follow-up** |
|   **Figure 13 - Proportional odds for Item 7 at 8-weeks follow-up** |
|   **Figure 14 - Proportional odds for Item 7 at 12-months follow-up** |
|   **Figure 15 - Proportional odds for Item 8 at 8-weeks follow-up** |
|   **Figure 16 - Proportional odds for Item 8 at 12-months follow-up** |
|   **Figure 17 - Proportional odds for Item 9 at 8-weeks follow-up** |
|   **Figure 18 - Proportional odds for Item 9 at 12-months follow-up** |
|   **Figure 19 - Proportional odds for Item 10 at 8-weeks follow-up** |
|   **Figure 20 - Proportional odds for Item 10 at 12-months follow-up** |
|   **Figure 21 - Proportional odds for Item 11 at 8-weeks follow-up** |
|   **Figure 22 - Proportional odds for Item 11 at 12-months follow-up** |
|   **Figure 23 - Proportional odds for Item 12 at 8-weeks follow-up** |
|   **Figure 24 - Proportional odds for Item 12 at 12-months follow-up** |


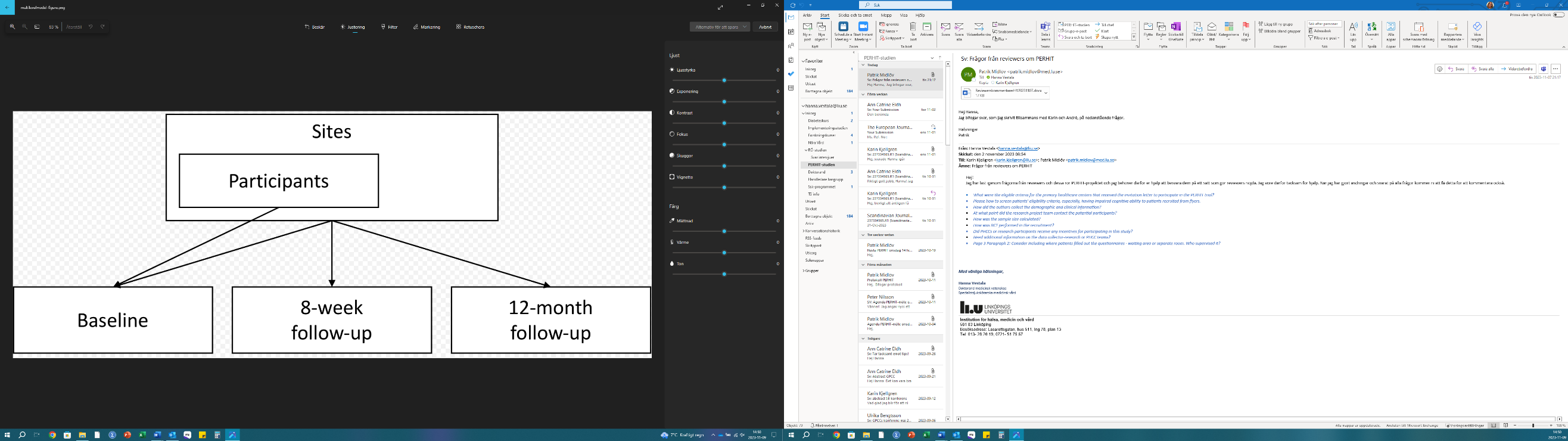


**Figure 25: Depiction of the multilevel model**
